# Whole genomic sequencing based genotyping reveals a specific X3 sublineage restricted to Mexico and related with multidrug resistance

**DOI:** 10.1038/s41598-020-80919-5

**Published:** 2021-01-21

**Authors:** Ana Cristina Jiménez-Ruano, Carlos Francisco Madrazo-Moya, Irving Cancino-Muñoz, Paulina M. Mejía-Ponce, Cuauhtémoc Licona-Cassani, Iñaki Comas, Raquel Muñiz-Salazar, Roberto Zenteno-Cuevas

**Affiliations:** 1 Programa de Maestría en Ciencias de la Salud, Instituto de Ciencias de la Salud, Universidad Veracruzana, Xalapa, Veracruz México; 2 Instituto de Salud Pública, Universidad Veracruzana, Av. Luis Castelazo Ayala S/N, A.P. 57, Col. Industrial Animas, 91190 Xalapa, Veracruz México; 3 Biomedical Institute of Valencia IBV-CSIC, Valencia, Spain; 4 Tecnologico de Monterrey, School of Engineering and Sciences, Monterrey, Nuevo Leon Mexico; 5 CIBER of Epidemiology and Public Health, Madrid, Spain; 6 Laboratorio de Epidemiología y Ecología y Molecular, Escuela de Ciencias de la Salud, Universidad Autónoma de Baja California, Ensenada, Baja California México; 7 Red Multidisciplinaria de Investigación en Tuberculosis, Mexico City, Mexico

**Keywords:** Microbiology, Molecular biology

## Abstract

Whole genome sequencing (WGS) has been shown to be superior to traditional procedures of genotyping in tuberculosis (TB), nevertheless, reports of its use in drug resistant TB (DR-TB) isolates circulating in Mexico, are practically unknown. Considering the above the main of this work was to identify and characterize the lineages and genomic transmission clusters present in 67 DR-TB isolates circulating in southeastern Mexico. The results show the presence of three major lineages: L1 (3%), L2 (3%) and L4 (94%), the last one included 16 sublineages. Sublineage 4.1.1.3 (X3) was predominant in 18 (27%) of the isolates, including one genomic cluster, formed by eleven multidrug resistant isolates and sharing the SIT 3278, which seems to be restricted to Mexico. By the use of WGS, it was possible to identify the high prevalence of L4 and a high number of sublineages circulating in the region, also was recognized the presence of a novel X3 sublineage, formed exclusively by multidrug resistant isolates and with restrictive circulation in Mexico for at least the past 17 years.

## Introduction

According to the WHO, ten million people in the world became ill with tuberculosis (TB) in 2018, around 15% of which died from the disease^1^. In Mexico, an estimated 29,000 cases of TB occurred in 2018, of which almost 3% were new and 11% were previously treated cases that presented rifampicin-(RR-TB) and multidrug- (MDR-TB) resistance^1^.

Whole genome sequencing (WGS) of TB isolates has multiple advantages over traditional genotyping techniques (MIRU-VNTR and spoligotyping) since it allows more robust *Mycobacterium tuberculosis* complex (MTBC) classification into lineages and sublineages, through the identification of a panel of 62 single-nucleotide polymorphisms (SNPs)^2^. The WGS genotyping method facilitates understanding of the dynamics of the disease and enables the implementation of measures focused on containment of the specific types of TB genotypes circulating within a given region^2–4^. Moreover, WGS has a greater power of discrimination and resolution of single nucleotide differences between clinical isolates. This information is extremely useful for the establishment of genetic relationships and identification of relationship levels between strains, which can help determine the formation of a genomic cluster, i.e., of cases derived from recent transmission^4,5^. The WGS can also be used as a tool for epidemiological surveillance and control of TB transmission, since it allows a more accurate description of the etiology of an outbreak and better resolution of the transmission topology, enabling definition of the directionality and evolution of transmission^4,6,7^.

The studies related to TB genotyping in Mexico using traditional methodologies such as MIRU-VNTR and spoligotyping, have shown a high diversity of circulating lineages; T (20%), X (11%), LAM (6%), EAI (7%), H (3%) and, to a lesser extent, S (1%) and Beijing (1%)^8^. However, an important proportion (20–60%) of the isolates so far characterized are frequently defined as orphans or are misclassified^8–11^. This lack of information greatly limits the potential use of genotyping techniques to establish clear inferences regarding the lineages in circulation and the design of epidemiological and public health measures. The aim of this study was therefore to identify and characterize, through analysis of WGS, the lineages and genomic transmission clusters present in DR-TB isolates circulating in southeastern Mexico.

## Materials and methods

### Population

This is a descriptive cross-sectional study, which included 67 genomes of *Mycobacterium tuberculosis* complex (MTBC) from patients diagnosed with pulmonary TB in the state of Veracruz, Mexico. The MTBC strains were randomly selected from the drug resistant strains bank of the Public Health Institute of Veracruz, including isolates recovered from 2013 to 2016.

MTB strains were isolated in LJ media, and the phenotypic drug sensitivity test (DST) against first-line drugs was performed using the fluorometric method (BACTEC, MGIT 960 Becton–Dickinson), according to standard conditions: isoniazid (H) > 0.1 μg/mL, rifampin (R) 1.0 μg/mL, ethambutol (E) 5.0 μg/mL and streptomycin (S) 1.0 μg/mL. Pyrazinamide sensitivity was determined using a BACTEC MGIT 960 PZA kit (Becton Dickinson).

### DNA extraction and WGS

Genomic DNA was extracted and purified following the CTAB method as previously described^12^. The DNA was quantified using a nanodrop (ThermoScientific, USA), with subsequent adjustment to a concentration of 0.2 ng/µL. The WGS libraries were prepared according to Nextera XT (Illumina, CA., USA) protocol, using 1 ng of DNA previously quantified by Qubit fluorometer (Invitrogen, CA, USA). Quality control of the genomic libraries was determined using TapeStation (Agilent Genomics), which was normalized and sequenced using NexSeq 500 (Illumina, CA., USA) in a 2 × 150 paired-end format.

### Bioinformatics analysis

Given the potential presence of contaminant DNA not corresponding to MTBC, the Kraken software V2^13^ was first used to classify the WGS reads. Further focus was directed only at those reads that belonged to MTBC species^14^. The WGS analysis, including mapping and variant calling (SNP and INDELS), was performed following a previously reported pipeline^7,15^, which has been described, validated and available online at http://tgu.ibv.csic.es/?page_id=1794. Variants that were present in at least 20 reads and at ≥ 90% of frequency within each isolate were called fixed-SNP (used to detect phylogenetic mutations). Unlike, variants in at least ten reads at ≤ 10% frequency called no fixed-SNP (used to detect antibiotic resistance). The analysis of the polymorphisms related to each anti-tuberculosis drug has been previously described^16^.

### Phylogenetic analysis, genotyping and identification of genomic transmission clusters

In order to build a phylogeny as well as to identify the genomic transmission, a concatenated alignment was created with the fixed-SNP of all clinical isolates. This alignment consisted in 7596 non-redundant positions. This alignment was used to infer phylogeny using the maximum likelihood phylogenetic approach implemented in MEGA V6^17^, applying a general time-reversible model of nucleotide substitution, with a gamma distribution (GTR + GAMMA) and considering 1000 bootstraps. The tree was visualized in iTOL v. 4^18^.

Moreover, this concatenated fixed-SNP alignment was used to identify the genomic clusters that reveal transmission events. We calculated pairwise genetic distances between each clinical isolate using “ape” R library. A ≤ 12 SNP threshold was applied to delineate the genomic clusters, as proposed by proposed by Walker et al.^5^.

Strains were classified according to the presence of 62 phylogenetic variants associated with lineages and sublineages, as proposed by Coll et al.^2^.

### Identification of specific SNPs in the transmission clusters

This analysis was performed using the previously described pipeline^7,15^, according to the following procedure: (1) development of a SNP-call in the 67 isolates that comprise the sample; (2) selection of the variants found in the cluster with the largest number of isolates (C1); (3) comparison of the list of specific SNPs found in the clusters with a global collection of 300,000 SNPs in order to remove SNPs related with homoplasy, SNPs shared with strains from other countries, and mutations related to lineage assignment and drug resistance; and (4) distinction of resulting SNPs by type, essential or non-essential activity of the gene, and identification of synonymous and non-synonymous variants, for generation of the final list.

### In silico spoligotyping using WGS

The in silico spoligotyping using the WGS reads was conducted using the program SpoTyping V 2.0, according to the authors instructions^19^. The binary code obtained for each isolate was analyzed with the SITVIT2 platform at http://www.pasteur-guadeloupe.fr:8081/SITVIT2/^20^, in order to identify the sublineage and assign the respective spoligotype international type (SIT).

### Analysis of association between patient variables, lineages and genomic transmission clusters

Information related to the clinical, sociodemographic and geographic location (jurisdiction) of patients at the time of diagnosis was obtained from laboratory records and anonymized. Association between variables was determined based on the calculation of odds ratio (OR), considering a 95% confidence interval (IC 95). Calculation of the Fisher's test was performed considering a value of *p* < 0.05 as significant in terms of establishing association between variables. All calculations were performed using the software SPSS V.12.

### Ethics statements

Informed consent was waived since the study did not involve direct contact with patients. This waived and all aspects related with this research were approved by the Ethics Committee of the Health Science Institute of the University of Veracruz, protocol number: 30CEI00120180131-007/2019. All experimental protocols in this study were performed in accordance with the relevant guidelines and regulations.

## Results

### Population

Sixty-seven patients were included in the study: the average age was 45.4 ± 14.6 years, with males forming the most prevalent population at 39 (58%) individuals. In terms of comorbidities, 38 (57%) patients presented type 2 diabetes mellitus (T2DM), two (3%) had an HIV infection, seven (11%) presented alcoholism and eight (12%) malnutrition. A total of 29 patients (43%) were classified as new cases of TB, while the rest were relapses, readmissions or retreatments. Shortened primary treatment was applied to 31 patients (46%), while individualized retreatment was used in 16 (24%).

**Figure 1:**
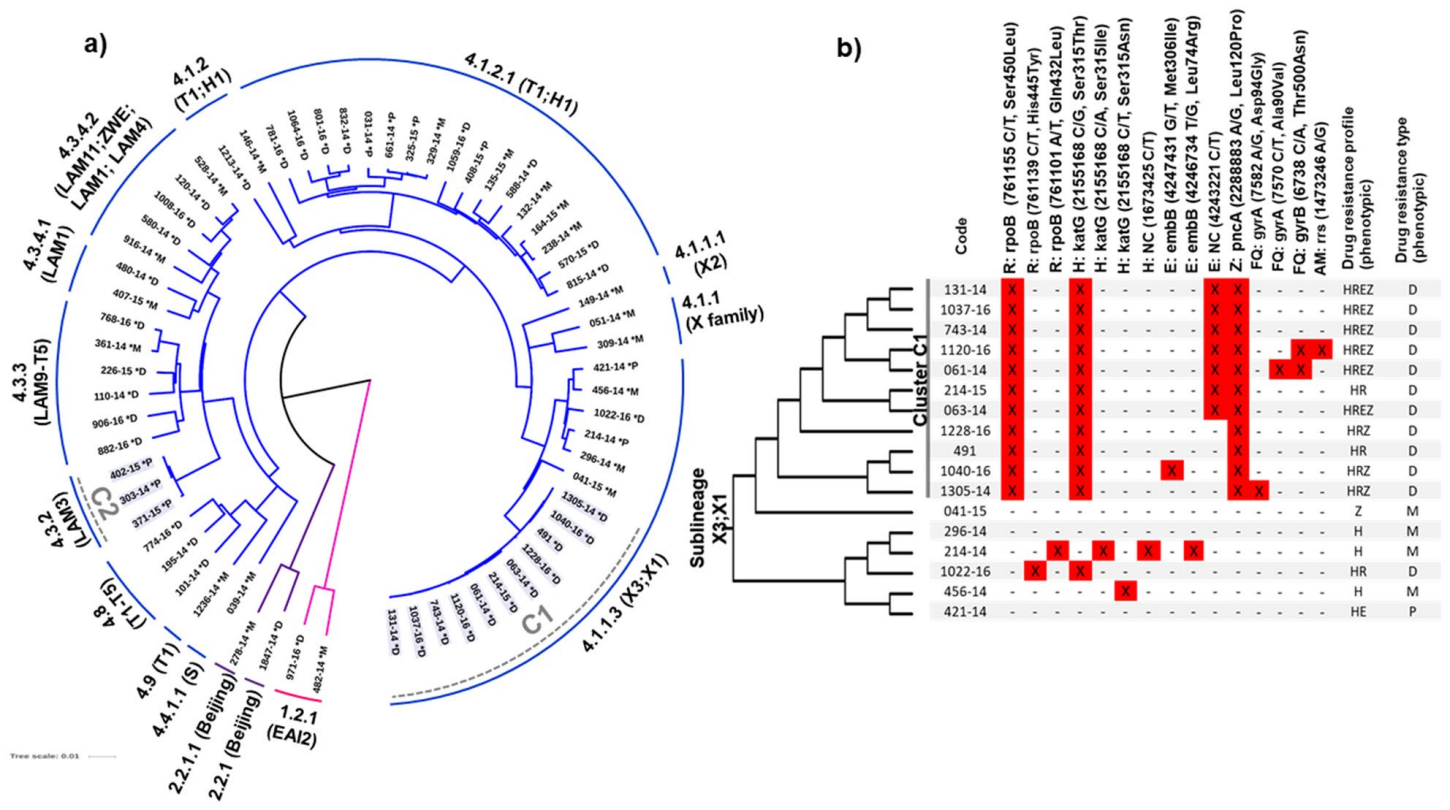
(**a**) Phylogenetic tree, using the maximum likelihood method, of isolates of drug resistant TB. (**b**) Comparison of mutations that confer resistance to drugs in isolates from sublineage 4.1.1.3 (X3). *M* mono-resistance, *P* poly drug resistance, *D* multidrug resistance, *H* isoniazid, *R* rifampicin, *E* ethambutol, *Z* pyrazinamide, *FQ* fluoroquinolones, *AM* aminoglycoside. (**a**) Tree was made using the freely version of iTOL V.4 (https://itol.embl.de/).

According to DST the 67 isolates showed resistance to at least one first-line drug (Fig. 1a), 40 (60%) were resistant to rifampicin (R), 51 (76%) to isoniazid (H), 16 (24%) to ethambutol (E) and 23 (34%) to pyrazinamide (Z). Mono-resistance was observed in 22 (33%) isolates, poly-drug resistance in nine (13%) and 36 (54%) were MDR-TB.

### Genotyping, phylogenomic and identification of genomic transmission clusters

All samples were successfully sequenced. The number or reads varied from 334,031 to 4,391,976. At least the 88% of the reference genome was covered and the average coverage depth of all isolates was 138.63 (ranging from 19 to 269, median 141). Detailed sequencing information of samples analyzed is in Supplementary Table S2.

Three lineages were identified: L1 (Indo-Oceanic) and L2 (East Asian/Beijing), both with two isolates each (3%), and L4 (Euro-American) as the most abundant, including 63 (94%) isolates distributed among thirteen sublineages, the main sublineages were L4.1.1.3 (X3) with 18 (27%) isolates, followed by sublineage L4.1.2.1 (T1;H1) with 17 (25%) strains and L4.3.3 (LAM9;T5) with six (9%) isolates (Table 1 and Fig. 1a).

**Table 1:** Linages and sublineages found in isolates of drug resistant TB from Veracruz, Mexico.

Lineage	Sublineage		n	%
1. Indo-Oceanic (3%)	1.2.1	EAI2	2	3
2. East-Asian (3%)	2.2.1	Beijing	1	1.5
	2.2.1.1	Beijing	1	1.5
4. Euro-American (94%)	4.1	T; H; X	1	1.5
	4.1.1	X family	2	3
	4.1.1.1	X2	1	1.5
	4.1.1.3	X3:X1	18	26.9
	4.1.2	T1; H1	3	4.5
	4.1.2.1	T1; H1	17	25.4
	4.3.2	LAM3	3	4.5
	4.3.3	LAM9; T5	6	9
	4.3.4.1	LAM1	2	3
	4.3.4.2	LAM11-ZWE; LAM9; LAM1; LAM4	5	7.5
	4.4.1.1	S	1	1.5
	4.8	T1; T2; T3; T4; T5	3	4.5
	4.9	T1	1	1.5
		Total	67	100

The occurrence of only two genomic transmission clusters was observed; cluster “C1”, based on a threshold of < 12 SNPs, is composed of eleven MDR isolates and belongs to sublineage L4.1.1.3 (X3;X1), and cluster “C2”, with three poly-resistant isolates located in sublineage L4.3.2 (LAM3) (Fig. 1a).

Figure 1b shows the mutation patterns related to drug resistance in the 17 isolates forming the L4.1.1.3 sublineage and clearly shows the differences in the mutation profiles between the eleven isolates that make up C1 (131-14, 1037-16, 743-14, 1120-16, 061-14, 214-15, 063-14, 1228-16, 491-14, 1040-16 and 1305-14) and the reaming isolates located outside this cluster. In addition, two isolates from C1, mutations associated with resistance to fluoroquinolones, strain 061-14 had the change Thr500Asn at *gyrB*, and 1305-15 the mutation Asp94Gly at *gyrA* gene, predicting a pre-extensive drug resistant profile (pre-XDR-TB) (Fig. 1b). A third isolate, also from C1 (1120-16), showed mutations Thr500Asn at *gyrB.* These mutations are associated with resistance to fluoroquinolones and aminoglycosides, which could potentially be considered an XDR- TB isolate (Fig. 1b). Finally, the isolates included in C2 only showed one mutation, G/C, at genomic position 2155168, given place to the change Ser315Thr (nucleotide position 1280) in the *katG* gene.

### Identification of specific SNPs in cluster C1 (X3)

Excluding those SNPs associated with lineage and antibiotic resistance-related genes, a subset of 83 SNPs was found specifically in isolates belonging to the C1 cluster and with an X3 sublineage (Supplementary Table S1), of which 26 were identified as synonymous. It is important to highlight that the remaining strains within the X3 sublineage that do not belong to C1:X3, do not share these exclusive SNPs (Fig. 1b).

Functional annotation indicates that eight SNPs were detected in non-coding regions, 19 SNPs were found in hypothetical proteins, including six synonymous variants. 56 SNPs were found in the same number of genes with a specific function. These were divided into: 19 SNPs in genes with essential activity, including four synonymous variants, and 37 SNPs in the same number of genes with nonessential activity, which included 16 synonymous variants. Of the 56 variants found in genes with a known function, nine non-synonymous variants were found in genes associated with virulence; *pks16, Ace, mmpL7, devS, mmpL3, pks6, mas, fadD22 and dacB*; one gene was associated with transmission, *emrB*, three SNPs were found in genes related to drug resistance, *embA, amiB2,* and *dnaE2*, and five were found in genes associated with resistance and virulence, *pks16, mmpl7, mmpL3, pks6* and *pks4* (Supplementary Table S1).

### In silico spoligotyping

A total of 38 spoligotype patterns were detected. Table 2 shows the octal codes, SITs and lineages identified by the in silico spoligotyping analysis conducted in the 67 genomes. A total of 54 (81%) isolates were assigned to a respective SIT and lineage, four (6%) had only a SIT assignation, and nine (13%) of isolates were classified as orphans.

**Table 2:** Genotypic characterization by in silico spoligotyping and phylogenetic variants of DR-TB circulating in Veracruz, Mexico.

Isolate	In silico spoligotyping				WGS lineage SNPs assignment	
	Octal code	Lineage	SIT	Sublineage	Code	Spol-correspondence
1847-14	000000000003771	Beijing (3%)	1	Beijing	2.2.1	Beijing
278-14					2.2.1.1	Beijing
971-16, 482-14	677777477413771	EAI (3%)	19	EAI2-Manila	1.2.1	EAI2
1004-13	777777760020611	H (7.5%)	948	H3	4.1.2	T1; H1
146-14	777777774020611		2642	H1		
329-14, 801-16	000000004020771		2	H2	4.1.2.1	T1; H1
570-15	777777774020771		47	H1		
361-14^a^, 110-14	777777607760771	LAM (13.4%)	42	LAM9	4.3.3	LAM9; T5
226-15^a^	637777607760771		578	LAM1	4.3.3	LAM9; T5
407-15	677737607760771		17	LAM2	4.3.4.1	LAM1
480-14	677777607760771		20	LAM1		
120-14	777777607760771		42	LAM9	4.3.4.2	LAM11-ZWE; LAM9; LAM4
1008-16	777777607760731		60	LAM4		
580-14^a^	777577607760771		1535	LAM9		
916-14	577737607760771		3019	LAM5		
039-14	776377777760771	S (1.5%)	34	S	4.4.1.1	S
1213-14	637777777760731	T (25.4%)	–	T2	4.1.2	T1; H1
815-14	757777777760771		154	T1	4.1.2.1	T1; H1
832-14	777777777740771		172	T1		
135-15^a^	777737777760771		37	T3		
325-15	777777775760771		122	T1		
164-15, 238-14, 132-14, 588-14, 661-14, 031-14, 1064-16	777777777760771		53	T1		
768-16	777777677760771		291	T1	4.3.3	LAM9; T5
195-14, 774-16	777777777760771		53	T1	4.8	T1; T2; T3; T4; T5
101-14	000000177760771		258	T		
1236-14^a^	777777777760700		51	T1	4.9	T1
051-14, 309-14, 1022-16, 456-14, 421-14	777776777760771	X (26.9%)	119	X1	4.1.1	X
041-15	700076777760771		92	X3	4.1.1.3	X3; X1
1040-16, 491, 1305-14, 1228-16, 061-14, 1120-16, 063-14, 214-15, 131-14, 1037-16, 743-14	700076717760771		3278	X3		
149-14	777776777760601		137	X2	4.1.1.1	X2
906-16	777774077560771	No lineage	222	–	4.3.3	LAM9; T5
404	777776770000000		450	–	4.1	T; H; X
214-14, 296-14				–	4.1.1.3	X3; X1
454^a^	700076707760771	Orphans	–	–	4.1.1.3	X3; X1
408-15^a^	701777777760771		–	–	4.1.2.1	T1; H1
1059-16^a^	777677404760571		–	–		
781-16^a^	777770345760771		–	–		
303-14^a^, 402-15^a^, 371-15^a^	376173607760771		–	–	4.3.2	LAM3
528-14^a^	777770003760771		–	–	4.3.4.2	LAM9, LAM11-ZWE
882-16^a^	777777660000131		–	–	4.3.3	LAM9; T5

^a^Described for the first time in Mexico.

Three main lineages were recorded by the in silico spoligotyping analysis: EAI (L1) and Beijing (L2), with two (3%) isolates each, and Euro-American as the predominant lineage that included 50 (75%) of isolates and considered four sublineages; X including eighteen (26%) of the isolates distributed among four spoligotyping patterns, T with seventeen (25%) of the isolates among ten spoligotyping patterns, LAM with 13% (9) of the isolates among eight spoligotyping patterns, and H with 7% (5) of the isolates in four spoligotyping patterns.

The most abundant SITS were: (1) SIT 3278 (700076717760771), present in eleven (16%) isolates. (2) SIT 53 (7777777760771), was found in nine (13%) of the isolates and (3) SIT 119 (777776777760771), found in five (7%) isolates (Table 2).

Comparison between the lineages and sublineages assigned by in silico spoligotyping and those defined by WGS phylogenetic variant analysis presented a coincidence of 70% (Table 2). The main differences were those isolates identified with a SIT but no lineage, and those identified as orphans, which were correctly assigned using WGS analysis (Table 2).

### Associations between patient variables, lineages and genomic transmission clusters

No significant association was observed between sublineages and most of the variables recovered. Only one risk association was found, between male sex and development of TB with the X3 sublineage where an OR of 3.8 was observed (IC 1.1–13,), however, the *p* value was limited (*p* = 0.053).

In addition, four statistically significant associations were observed with C1 (Table 3): (1) development of a TB infection with a strain from C1 and presenting MDR-TB (*p* = 0.0005), unfortunately, the presence of empty cells made it impossible to calculate the OR value; (2) a protective association between residence in the northern part of the state of Veracruz and development of TB with a strain from C1 (*p* = 0.026); however, it was impossible to perform the respective OR analysis; (3) an association between residence in the central region of the state and development of TB with a strain from C1 (*p* = 0.039), with an OR of 8.3 (IC 95% 1–69.6); and (4) an association between residence in the region of Xalapa (capital city of the state) and development of TB with a strain from C1 (*p* = 0.04), with an OR of 5.7 (IC 95% 1.2–26.5). This illustrates the importance of place of residence, and specifically residence in the capital city (Xalapa), as a risk factor in acquisition of a TB infection with a strain from C1.

**Table 3:** Variables with significant association with isolates from the C1 cluster.

Independent variable	Dependent variable	Case %	Control %	OR^a^	IC 95%	Fisher’s exact test**
Isolates in C1^a^	MDR condition	30.6 (11/36)	0 (0/31)	–	–	*p* = 0.0005
Isolate not in C1^a^		69.4 (25/36)	100 (31/31)	1.0	–	–
Sanitary jurisdiction from northern zone (II, III, IV and XII)^a^	Isolates from C1 cluster	0 (0/11)	36.4 (20/55)	–	–	*p* = 0.026
Sanitary jurisdiction from central and southern zone^a^		100 (11/11)	63.6 (35/55)	1.0	–	–
Sanitary Jurisdiction from central zone (V, VI, VII, VIII and IX)		90.9 (10/11)	54.5 (30/55)	8.3	1.1–69.6	*p* = 0.039
Sanitary jurisdiction from northern and southern zone^a^		9.1 (1/11)	45.5 (25/55)	1.0	–	–
Sanitary jurisdiction of Xalapa (V)		36.4 (4/11)	9.1 (5/55)	5.7	1.2–26.5	*p* = 0.04
Sanitary jurisdiction other than Xalapa (I–IV, VI–XII)^a^		63.6 (7/11)	90.9 (50/55)	1.0	–	–

**p value < 0.05 was considered significant.

^a^OR calculation was impossible to determine.

## Discussion

According to the WHO, 24,096 new TB cases were reported in Mexico in 2018, of which less than 3% of the new, and 11% of the previously treated, cases presented resistance to rifampicin, as well as multi-drug resistance^1^. Nevertheless, 29 (43%) of the DR-TB isolates recovered and analyzed in this study came from individuals classified as new cases, remaining 38 (57%) were relapses, readmissions or retreatments, of which 36 (54%) were classified as MDR-TB. This figure shows the burden of DR-TB in the region.

This study assigned lineages and sublineages to 100% of the isolates analyzed, something never achieved in previous studies in Mexico using conventional techniques such as MIRU and spoligotyping^8,9,21,22^. This demonstrates the higher resolution of WGS compared to traditional genotyping methods, as previously described^2–7,23,24^.

Three major lineages were identified (L1, L2 and L4), with 16 sublineages. L4 presents the highest proportion of isolates (94%) and sublineages (13). This diversity of lineages concurs with previous studies and confirms the predominance of isolates with the Euro-American lineage (L4) in the country^8–10,22,25,26^ and also in countries from center and South America^27,28^. This success of the transmission has been explained as a consequence of the European colonization in the fifteen and sixteen centuries, with importation of sub-lineages^15,27,29,30^, and also in terms of its adaptation to the immune response of the host in the different locations^31^.

Only 21% of isolates were clustered by WGS analysis, while the use of traditional genotyping techniques in isolates from Mexico has produced percentages of clustering that range from 60 to 70%^8,10,25,32–34^. The overestimation of clustering by traditional genotyping techniques is well recognized, particularly in the case of spoligotyping, whereas WGS has a better clustering discrimination capability and provides better descriptions of transmission clusters that occur in the population, in addition, the inclusion of information from patients is of great help to identify epidemiological links and transmission routes in patients located within identified clusters^4,7,35,36^. Furthermore, L4 genetic clusters detected by MIRU-VNTR have been described as overestimated^37^. Undoubtedly, genotyping and phylogenomic analysis by WGS will have important implications for the genotypic and epidemiological analysis of TB in Mexico.

The most frequent L4 MTBC sublineages were L4.1.1 (X) with 21 (31%) isolates, followed by L4.1.2 (T) with 20 (30%) isolates. The T sublineage is frequently described in Mexico^8,21^, while X has a very low occurrence and has only been described with prevalences ranging from 21 to 29% in two reports from the central and northern regions of the country^11,34^. The high proportion found here is therefore unusual and is the first such finding for isolates circulating in the southern region of the country. However, the relatively small number of isolates included in this study must be taken into account. Considering the above, the incorporation of a greater number of isolates, recovered through an epidemiological-genomic surveillance system^38^, will allow a clearer definition of the lineages/sub lineages circulating in the region, and even identify imported cases derived from migration effects.

A significant association was identified between cluster C1 (X3) and geographical location, and this was most significant in the region of Xalapa, the capital city of the state. Previous studies in this region have shown similar associations between other lineages such as H2 and a specific region in the north of the state of Veracruz (Tuxpan)^10^. These results describe the preferential distribution of certain genotypes in specific geographic regions of the state, and illustrate the value of this analysis in terms of identifying prevalence and the transmission routes of specific lineages, information of importance for the adequate design of specific preventive strategies.

All isolates included in C1 (X3) were found to be MDR-TB and shared almost the same polymorphisms pattern related to resistance against rifampicin, isoniazid and pyrazinamide, with the exception of three isolates (1228-06, 481-14, 1305-14) that were phenotypically susceptible to ethambutol and lacked any of the common mutations frequently found in genes associated with resistance to this drug, apart from isolate 1040-16 that had the mutation G/T at genomic position 4247431, given place to change Met306Ile, nucleotide position 918, in the *embB gene*.

These data suggest that resistance to ethambutol has arisen in a second moment of transmission of this strain. Finally, two of the isolates showed SNPs that confer a potential pre-XDR-TB and XDR-TB condition, confirming the potential of WGS analysis as a tool for predicting drug resistance. This shows the evolution of this strain according to the interaction with their respective hosts.

In addition, we described the functional annotation in the 83 variants exclusively identified in the C1 isolates, from which nine non-synonymous variants were in genes implicated in the virulence of *M. tuberculosis,* one in transmission, two in genes related to drug resistance and five in genes with participation in both of these processes. Altogether, this information evidences the evolution and adaptation of this clone to this region of Mexico, with an increase in virulence and tendency to develop pre- and XDR-TB forms. Further studies that include isolates from different regions of Mexico are necessary in order to evaluate the impact of this clone in the epidemiology of DR-TB and the participation, not only of the non-synonymous variants found in the genes associated with resistance, virulence and transmission, but also of the remaining SNPs found in the isolates that form this apparently new X sublineage of TB.

An important volume of genotyping data has been accumulated using spoligotyping and MIRU-VNTR, which is an important issue when comparative phylogenomic data is obtained by WGS genotyping. The in silico spoligotyping and comparison with phylogenomic analysis showed a concordance close to 70%, with the main differences found in those isolates lacking a lineage or classified as orphans.

A detailed search of these spoligotypes in SITVIT2 and the author´s database confirms that more than 80% of the spoligotypes has been described previously in the country (Table 2). It was also observed that all of the members of C1 shared the same octal pattern (700076717760771) and SIT (3278, X3). This pattern, according to SITVIT2, has been previously described in a single isolate from Spain and in two isolates from Mexico recovered in 2003, one from the state of Colima and the other from Quintana Roo^33^ and, more recently, in five isolates from the state of Veracruz recovered in the period 2012–2013^10^. In both of these reports, the isolates were clustered and presented a phenotypic MDR-TB condition. This information confirms that this X3 sublineage is strongly associated with MDR-TB, is restricted to Mexico and has had national circulation over the entire country for at least 17 years, with recent growing expansion in the central setting of the state of Veracruz. Further WGS studies, including isolates from other states, are necessary in order to determine the level of expansion of this sublineage.

Perhaps the major limitations of this study were related to the restricted number of isolates analyzed, the failure to include fully susceptible isolates and the lack of phenotypic DST studies for second line drugs. Undoubtedly, there is an urgent need to increase the number of studies related to WGS analysis of tuberculosis in Mexico in order to identify in greater detail the diversity of the circulating lineages and the presence and extent of genomic transmission clusters in the various regions of the country, as well as to determine the variables that could function as risk factors of transmission.

We conclude that WGS was extremely useful in terms of defining the lineages in the totality of the isolates analyzed and also in the identification of genomic transmission clusters. It also identified the presence of a cluster comprising a MDR strain with an X3 sublineage, specifically located in the center of the state of Veracruz, but one that has been expanding in Mexico over the last 17 years. Further studies will be required in order to explain the origin of this strain, its transmission routes and its implications for public health.

## Supplementary Information

Supplementary Table S1.

Supplementary Table S2.

## Data Availability

The data underlying the genome sequences presented in this study are available at ENA: https://www.ebi.ac.uk/ena. Accession number: PRJEB30933.
