# Supplementary material for: Whole genomic sequencing based genotyping reveals a specific X3 sublineage restricted to Mexico and related with multidrug resistance
Source: Sci Rep. 2021 Jan 21;11:1870. doi: 10.1038/s41598-020-80919-5 (PMC7820219; doi:10.1038/s41598-020-80919-5)
Supplement: Supplementary file 1 — Supplementary Table S1. [file 41598_2020_80919_MOESM1_ESM.docx]

- **Whole genomic sequencing based genotyping reveals a specific X3 sublineage restricted to Mexico and related with multidrug resistance**
- Ana Cristina Jiménez-Ruano^1-2^, Carlos Francisco Madrazo-Moya^1,3^, Irving Cancino-Muñoz^3^, Paulina M. Mejía-Ponce^4^, Cuauhtémoc Licona-Cassani^4^, Iñaki Comas^3,5^, Raquel Muñiz-Salazar^6-7^, Roberto Zenteno-Cuevas^1,2,7^*
- 1. Programa de Maestría en Ciencias de la Salud, Instituto de Ciencias de la Salud. Universidad Veracruzana. Xalapa, Veracruz, México.
- 2. Instituto de Salud Pública, Universidad Veracruzana. Xalapa, Veracruz, México.
- 3. Biomedical Institute of Valencia IBV-CSIC, Valencia, Spain
- 4. Tecnologico de Monterrey, School of Engineering and Sciences. Monterrey, Nuevo Leon, Mexico
- 5. CIBER of Epidemiology and Public Health, Madrid, Spain
- 6. Laboratorio de Epidemiología y Ecología y Molecular, Escuela de Ciencias de la Salud, Universidad Autónoma de Baja California, Ensenada, Baja California, México
- 7. Red Multidisciplinaria de Investigación en Tuberculosis. [www.remitb.org](http://www.remitb.org)

| **Supplementary Table S1. Single nucleotide polymorphisms specific to C1:X3 isolates** | | | | | | | | | | | | | | | |
| --- | --- | --- | --- | --- | --- | --- | --- | --- | --- | --- | --- | --- | --- | --- | --- |
| **Genomic position** | **Allele** | | | **Gene** | |  | | | | | | | | | |
|  | **WT** | | **Mutation** | **starts** | **ends** | **AA** | **Gene alias** | | **Gene name** | | **Nucleotide change** | | | **AA change** | **Gene function** |
| **Non coding region (NCR) n=8** | | | | | | | | | | | | | | | |
| 99494 | T | | G | 99251 | 99683 | NA | - | | IG91_Rv0090-Rv0091 | | | - | | - | - |
| 580772 | T | | A | 580582 | 580808 | NA | - | | IG495_Rv0490-Rv0491 | | | - | | - | - |
| 960223 | C | | T | 960152 | 960341 | NA | - | | IG877_Rv0861c-Rv0862c | | | - | | - | - |
| 1457547 | A | | G | 1457543 | 1457557 | NA | - | | IG1322_Rv1300-Rv1301 | | | - | | - | - |
| 2516716 | C | | T | 2516549 | 2516786 | NA | - | | IG2274_Rv2242-Rv2243 | | | - | | - | - |
| 2641242 | C | | G | 2641065 | 2641245 | NA | - | | IG2396_Rv2357c-Rv2358 | | | - | | - | - |
| 3595492 | G | | C | 3595434 | 3595712 | NA | - | | IG3272_Rv3218-Rv3219 | | | - | | - | - |
| 3880867 | C | | T | 3880654 | 3880906 | NA | - | | IG3521_Rv3462c-Rv3463 | | | - | | - | - |
| **Hypothetical protein (n=19). Missense variants (n=13)** | | | | | | | | | | | | | | | |
| 29750 | A | | G | 29722 | 31068 | 448 | - | | Rv0026 | | 29A>G | | | H10R | hypothetical protein |
| 545780 | A | | C | 545375 | 545821 | 148 | - | | Rv0455c | | 42T>G | | | F14L | hypothetical protein |
| 901807 | T | | C | 901635 | 902024 | 129 | - | | Rv0807 | | 173T>C | | | V58A | hypothetical protein |
| 1008951 | G | | A | 1008944 | 1010062 | 372 | - | | Rv0906 | | 8G>A | | | R3H | hypothetical protein |
| 1520572 | C | | T | 1520005 | 1521876 | 623 | - | | Rv1354c | | stop_gained | | | 1305G>A | hypothetical protein |
| 1574701 | C | | T | 1574510 | 1574767 | 85 | - | | Rv1398c | | 67G>A | | | E23K | hypothetical protein |
| 1819507 | G | | C | 1818575 | 1820029 | 484 | - | | Rv1619 | | 933G>C | | | E311D | hypothetical protein |
| 1856849 | G | | A | 1856774 | 1857724 | 316 | - | | Rv1647 | | 76G>A | | | D26N | hypothetical protein |
| 2487130 | C | | T | 2486994 | 2487416 | 140 | - | | Rv2219A | | 287G>A | | | G96D | hypothetical protein |
| 3014772 | G | | A | 3014663 | 3014965 | 100 | - | | Rv2699c | | 194C>T | | | P65L | hypothetical protein |
| 3653473 | C | | A | 3653448 | 3654632 | 394 | - | | Rv3272 | | 26C>A | | | P9Q | hypothetical protein |
| 4383745 | C | | G | 4383653 | 4383985 | 110 | - | | Rv3898c | | 241G>C | | | A81P | hypothetical protein |
| 3207329 | T | | G | 3206431 | 3207942 | 503 | - | | Rv2897c | | 614A>C | | | E205A | hypothetical protein |
| **Synonymous variants (n=6)** | | | | | | | | | | | | | | | |
| 1192822 | C | | T | 1192510 | 1194273 | 587 | - | | Rv1069c | | 1452G>A | | | V484V | hypothetical protein |
| 2339605 | A | | G | 2338709 | 2340874 | 721 | - | | Rv2082 | | 897A>G | | | P299P | hypothetical protein |
| 2982914 | A | | G | 2982699 | 2982980 | 93 | - | | Rv2665 | | 216A>G | | | L72L | hypothetical protein |
| 3327707 | C | | G | 3327023 | 3327736 | 237 | - | | Rv2972c | | 30G>C | | | S10S | hypothetical protein |
| 3858802 | C | | T | 3858259 | 3859662 | 467 | - | | Rv3439c | | 861G>A | | | P287P | hypothetical protein |
| 4343165 | G | | A | 4342770 | 4343321 | 183 | - | | Rv3867 | | 396G>A | | | L132L | hypothetical protein |
| **Genes with essential activity (n=19).** **Missense variants (n=15)** | | | | | | | | | | | | | | | |
| 202279 | C | | A | 200932 | 202479 | 515 | mce1C^1^ | | Rv0171 | | 1348C>A | | | P450T | MCE-family protein MCE1C |
| 208322 | G | | T | 207452 | 208420 | 322 | - | | Rv0176 | | 871G>T | | | A291S | mce associated trans membrane protein |
| 512091 | C | | T | 510702 | 515321 | 1539 | ctpH | | Rv0425c | | 3231G>A | | | M1077I | metal cation transporting P-type ATPase CtpH |
| 517819 | T | | A | 517803 | 518396 | 197 | def | | Rv0429c | | 578A>T | | | D193V | peptide deformylase |
| 645031 | T | | C | 644490 | 645470 | 326 | menC | | Rv0553 | | 542T>C | | | V181A | O-succinylbenzoate synthase |
| 1067050 | G | | T | 1066078 | 1067445 | 455 | - | | Rv0955 | | 973G>T | | | V325F | integral membrane protein |
| 1131937 | G | | A | 1131625 | 1133259 | 544 | pks16^1,2^ | | Rv1013 | | 313G>A | | | G105S | acyl-CoA synthetase |
| 1675278 | G | | T | 1675017 | 1676051 | 344 | hemH | | Rv1485 | | 262G>T | | | V88F | ferrochelatase |
| 1790452 | T | | G | 1790284 | 1791333 | 349 | bioB | | Rv1589 | | 169T>G | | | L57V | biotin synthase |
| 2514549 | A | | G | 2512539 | 2515244 | 901 | aceE^1^ | | Rv2241 | | 2011A>G | | | I671V | pyruvate dehydrogenase subunit E1 |
| 3060967 | C | | T | 3059855 | 3062506 | 883 | ftsK | | Rv2748c | | 1540G>A | | | D514N | cell division transmembrane protein FtsK |
| 3287806 | C | | T | 3285070 | 3287832 | 920 | mmpL7^1,2^ | | Rv2942 | | 2737C>T | | | L913F | trans membrane transport protein MmpL7 |
| 3498008 | C | | T | 3497529 | 3499265 | 578 | devS^1^ | | Rv3132c | | 1258G>A | | | E420K | two component sensor histidine kinase DEVS |
| 3498885 | A | | C | 3497529 | 3499265 | 578 | devS^1^ | | Rv3132c | | 381T>G | | | D127E | two component sensor histidine kinase DEVS |
| 4243920 | G | | A | 4243233 | 4246517 | 1094 | embA^2^ | | Rv3794 | | 688G>A | | | G230S | integral membrane indolylacetylinositol arabinosyltransferase |
| **Synonymous variants (n=4)** | | | | | | | | | | | | | | | |
| 1379313 | C | | T | 1378927 | 1379850 | 307 | sugA^1^ | | | Rv1236 | 387C>T | | G129G | | sugar-transport integral membrane protein ABC transporter SugA |
| 2482417 | T | | C | 2481965 | 2483626 | 553 | dlaT^1^ | | | Rv2215 | 453T>C | | V151V | | dihydrolipoamide acetyltransferase |
| 4034993 | G | | T | 4034352 | 4035710 | 452 | lpqF | | | Rv3593 | 642G>T | | V214V | | lipoprotein LpqF |
| 4274380 | C | | G | 4273739 | 4274593 | 284 | pirG | | | Rv3810 | 642C>G | | G214G | | exported repetitive protein precursor PirG (EXP53) |
| **Genes with nonessential activity (n=37). Missense variants (n=21)** | | | | | | | | | | | | | | | |
| 47734 | G | | A | 47366 | 48100 | 244 | - | | | Rv0043c | 367C>T | | | R123W | GntR family transcriptional regulator |
| 245080 | C | | G | 244484 | 247318 | 944 | mmpL3^1,2^ | | | Rv0206c | 2239G>C | | | G747R | transmembrane transport protein MmpL3 |
| 303595 | C | | A | 302866 | 305427 | 853 | nirB | | | Rv0252 | 730C>A | | | R244S | nitrite reductase large subunit |
| 488419 | G | | A | 485731 | 489939 | 1402 | pks6^1,2^ | | | Rv0405 | 2689G>A | | | G897R | membrane bound polyketide synthase |
| 524080 | C | | G | 522347 | 524533 | 728 | - | | | Rv0435c | 454G>C | | | A152P | putative ATPase |
| 685336 | G | | C | 685129 | 685926 | 265 | yrbE2A | | | Rv0587 | 208G>C | | | V70L | integral membrane protein YrbE2a |
| 877011 | C | | T | 876818 | 878440 | 540 | emrB^3^ | | | Rv0783c | 1430G>A | | | R477Q | integral membrane efflux protein EmrB |
| 1054725 | C | | T | 1054247 | 1055008 | 253 | - | | | Rv0945 | 479C>T | | | A160V | short chain dehydrogenase |
| 1410852 | C | | A | 1410431 | 1411819 | 462 | amiB2^2^ | | | Rv1263 | 422C>A | | | A141D | amidase |
| 1579534 | C | | G | 1577613 | 1579580 | 655 | priA | | | Rv1402 | 1922C>G | | | T641S | primosome assembly protein PriA |
| 1837613 | A | | G | 1837075 | 1839171 | 698 | uvrB | | | Rv1633 | 539A>G | | | D180G | excinuclease ABC subunit B |
| 1893672 | C | | G | 1893577 | 1894230 | 217 | - | | | Rv1667c | 559G>C | | | G187R | macrolide-transport ATP-binding protein ABC transporter |
| 2151789 | C | | G | 2151433 | 2152395 | 320 | aao | | | Rv1905c | 607G>C | | | G203R | D-amino acid oxidase |
| 3016539 | A | | G | 3015863 | 3016735 | 290 | suhB | | | Rv2701c | 197T>C | | | L66P | extragenic suppressor protein SuhB |
| 3089616 | C | | T | 3089045 | 3090361 | 438 | pepR | | | Rv2782c | 746G>A | | | R249H | zinc protease PEPR |
| 3279765 | T | | G | 3276380 | 3282715 | 2111 | Mas^1^ | | | Rv2940c | 2951A>C | | | D984A | multifunctional mycocerosic acid synthase membrane-associated MAS |
| 3299145 | C | | A | 3297837 | 3299954 | 705 | fadD22^1^ | | | Rv2948c | 810G>T | | | E270D | acyl-CoA synthetase |
| 3543138 | C | | A | 3542860 | 3544347 | 495 | - | | | Rv3175 | 279C>A | | | A93A | amidase |
| 3716782 | A | | G | 3715777 | 3716994 | 405 | dacB1 | | | Rv3330 | 1006A>G | | | T336A | penicillin-binding protein DacB1 |
| 3784559 | G | | A | 3781501 | 3784776 | 1091 | dnaE2^2^ | | | Rv3370c | 218C>T | | | A73V | error-prone DNA polymerase |
| 3868772 | G | | A | 3868352 | 3869755 | 467 | - | | | Rv3448 | 421G>A | | | A141T | integral membrane protein |
| 3990919 | A | | G | 3990771 | 3991523 | 250 | - | | | Rv3552 | 149A>G | | | D50G | CoA-transferase subunit beta |
| **Synonymous variants (n=16)** | | | | | | | | | | | | | | | |
| 1295499 | G | A | | 1294168 | 1296054 | 628 | typA | Rv1165 | | | 1332G>A | | K444K | | GTP-binding translation elongation factor TypA |
| 1317417 | G | A | | 1315234 | 1319982 | 1582 | pks4^1,2^ | Rv1181 | | | 2184G>A | | L728L | | polyketide beta-ketoacyl synthase PKS4 |
| 1756332 | C | T | | 1755445 | 1757310 | 621 | plsB1 | Rv1551 | | | 888C>T | | R296R | | glycerol-3-phosphate acyltransferase |
| 1768158 | C | G | | 1767135 | 1769432 | 765 | treY | Rv1563c | | | 1275G>C | | A425A | | maltooligosyltrehalose synthase TreY |
| 1841286 | C | A | | 1840572 | 1842242 | 556 | - | Rv1635c | | | 957G>T | | A319A | | transmembrane protein |
| 2006168 | C | T | | 2005161 | 2006447 | 428 | - | Rv1771 | | | 1008C>T | | A336A | | oxidoreductase |
| 2126293 | T | G | | 2125904 | 2127967 | 687 | - | Rv1877 | | | 390T>G | | G130G | | integral membrane protein |
| 2320529 | T | G | | 2317169 | 2320753 | 1194 | cobN | Rv2062c | | | 225A>C | | V75V | | cobaltochelatase |
| 2351083 | C | G | | 2349334 | 2352054 | 906 | helY | Rv2092c | | | 972G>C | | L324L | | ATP-dependent DNA helicase HelY |
| 2396221 | G | C | | 2396008 | 2396838 | 276 | uppP | Rv2136c | | | 618C>G | | P206P | | undecaprenyl pyrophosphate phosphatase |
| 2965673 | C | T | | 2965478 | 2965837 | 119 | - | Rv2640c | | | 165G>A | | P55P | | ArsR family transcriptional regulator |
| 3247874 | G | A | | 3245445 | 3251075 | 1876 | ppsA^1,2^ | Rv2931 | | | 2430G>A | | R810R | | phenolpthiocerol synthesis type-I polyketide synthase PPSA |
| 3268300 | G | A | | 3267737 | 3272203 | 1488 | ppsE^2,2^ | Rv2935 | | | 564G>A | | L188L | | phenolpthiocerol synthesis type-I polyketide synthase PPSA |
| 3369309 | C | T | | 3368823 | 3369854 | 343 | pfkA | Rv3010c | | | 546G>A | | G182G | | 6-phosphofructokinase |
| 4117513 | C | T | | 4117258 | 4118052 | 264 | - | Rv3677c | | | 540G>A | | R180R | | hydrolase |
| 1 Related to virulence , 2 Related to drug resistance, 3 Related to transmission | | | | | | | | | | | | | | | |
