# Supplementary material for: Whole genomic sequencing based genotyping reveals a specific X3 sublineage restricted to Mexico and related with multidrug resistance
Source: Sci Rep. 2021 Jan 21;11:1870. doi: 10.1038/s41598-020-80919-5 (PMC7820219; doi:10.1038/s41598-020-80919-5)
Supplement: Supplementary file 2 — Supplementary Table S2. [file 41598_2020_80919_MOESM2_ESM.docx]

- **Whole genomic sequencing based genotyping reveals a specific X3 sublineage restricted to Mexico and related with multidrug resistance**
- Ana Cristina Jiménez-Ruano^1-2^, Carlos Francisco Madrazo-Moya^1,3^, Irving Cancino-Muñoz^3^, Paulina M. Mejía-Ponce^4^, Cuauhtémoc Licona-Cassani^4^, Iñaki Comas^3,5^, Raquel Muñiz-Salazar^6-7^, Roberto Zenteno-Cuevas^1,2,7^*
- 1. Programa de Maestría en Ciencias de la Salud, Instituto de Ciencias de la Salud. Universidad Veracruzana. Xalapa, Veracruz, México.
- 2. Instituto de Salud Pública, Universidad Veracruzana. Xalapa, Veracruz, México.
- 3. Biomedical Institute of Valencia IBV-CSIC, Valencia, Spain
- 4. Tecnologico de Monterrey, School of Engineering and Sciences. Monterrey, Nuevo Leon, Mexico
- 5. CIBER of Epidemiology and Public Health, Madrid, Spain
- 6. Laboratorio de Epidemiología y Ecología y Molecular, Escuela de Ciencias de la Salud, Universidad Autónoma de Baja California, Ensenada, Baja California, México
- 7. Red Multidisciplinaria de Investigación en Tuberculosis. [www.remitb.org](http://www.remitb.org)

**Supplementary Table S2. Sequencing statistical information of samples analyzed**

| **Accesion number** | **Sample** | **Total Reads** | **Mean depth** | **Median depth** | **Coverage** | **% MTBC** |
| --- | --- | --- | --- | --- | --- | --- |
| ERR3148227 | 031_14S8 | 556184 | 29.765 | 29 | 0.9347 | 99.48 |
| ERR3148195 | 039_14S14 | 872231 | 103.92 | 106 | 0.9809 | 99.44 |
| ERR3148228 | 041_15S13 | 613964 | 35.069 | 35 | 0.9527 | 99.79 |
| ERR3148196 | 051_14S15 | 684420 | 81.442 | 83 | 0.9794 | 99.44 |
| ERR3148197 | 061_14S8 | 1127875 | 134.72 | 138 | 0.9816 | 99.68 |
| ERR3148198 | 063_14S4 | 1317213 | 157.75 | 162 | 0.9813 | 99.77 |
| ERR3148170 | 1008_16S10 | 3317090 | 207.93 | 217 | 0.9689 | 99.92 |
| ERR3148156 | 101_14S3 | 2948184 | 170.69 | 174 | 0.9717 | 99.29 |
| ERR3148171 | 1022_16S11 | 3307655 | 207.9 | 216 | 0.9765 | 99.91 |
| ERR3148172 | 1037_16S8 | 3640868 | 228.04 | 236 | 0.9776 | 99.92 |
| ERR3148173 | 1040_16S1 | 3704882 | 232.74 | 242 | 0.9773 | 99.91 |
| ERR3148226 | 1059_16S7 | 3788037 | 236.32 | 241 | 0.9783 | 99.71 |
| ERR3148174 | 1064_16S4 | 3465975 | 215.63 | 223 | 0.978 | 99.9 |
| ERR3148199 | 110_14S5 | 945301 | 112.67 | 115 | 0.9794 | 99.7 |
| ERR3148175 | 1120_16S2 | 3035509 | 191.11 | 198 | 0.9762 | 99.9 |
| ERR3148200 | 120_14S6 | 874057 | 104.16 | 107 | 0.9721 | 99.67 |
| ERR3148225 | 1213_14S3 | 3044663 | 187.47 | 186 | 0.9821 | 99.76 |
| ERR3148176 | 1228_16S12 | 3786207 | 236.96 | 246 | 0.9776 | 99.88 |
| ERR3148224 | 1236_14S1 | 2911855 | 175.22 | 172 | 0.9839 | 99.66 |
| ERR3148222 | 1305_14S12 | 3012397 | 189.29 | 194 | 0.9783 | 99.71 |
| ERR3148201 | 131_14S9 | 991239 | 112.39 | 115 | 0.984 | 95.05 |
| ERR3148157 | 132_14S5 | 2592655 | 151.71 | 158 | 0.9722 | 99.85 |
| ERR3148229 | 135_15S2 | 962371 | 114.65 | 119 | 0.9766 | 99.75 |
| ERR3148202 | 146_14S16 | 798468 | 94.779 | 97 | 0.979 | 99.58 |
| ERR3148203 | 149_14S18 | 895269 | 107.07 | 110 | 0.9753 | 99.7 |
| ERR3148158 | 164_15S12 | 459853 | 27.24 | 27 | 0.9373 | 99.77 |
| ERR3148182 | 1847_14S22 | 4278204 | 257.7 | 267 | 0.975 | 99.84 |
| ERR3148204 | 195_14S7 | 1015397 | 119.91 | 122 | 0.9817 | 99.62 |
| ERR3148159 | 214_14S9 | 922926 | 45.291 | 45 | 0.9524 | 93.27 |
| ERR3148160 | 214_15S3 | 425500 | 23.44 | 23 | 0.915 | 99.71 |
| ERR3148221 | 226_15S11 | 2352164 | 144.68 | 144 | 0.9754 | 99.31 |
| ERR3148205 | 238_14S10 | 1061351 | 121.07 | 124 | 0.9809 | 95.07 |
| ERR3148206 | 278_14S20 | 804066 | 94.855 | 97 | 0.9757 | 99.59 |
| ERR3148207 | 296_14S12 | 994926 | 119.42 | 123 | 0.9794 | 99.65 |
| ERR3148208 | 303_14S13 | 793224 | 94.394 | 96 | 0.9783 | 99.72 |
| ERR3148161 | 309_14S6 | 674568 | 36.885 | 36 | 0.9511 | 99.57 |
| ERR3148162 | 325_15S1 | 559196 | 31.346 | 32 | 0.9513 | 99.67 |
| ERR3148209 | 329_14S19 | 878146 | 104.41 | 107 | 0.9773 | 99.41 |
| ERR3148210 | 361_14S17 | 853895 | 101.42 | 103 | 0.9791 | 99.66 |
| ERR3148163 | 371_15S1 | 2341110 | 136.88 | 142 | 0.9688 | 99.82 |
| ERR3148211 | 402_15S1 | 1004801 | 119.89 | 121 | 0.9798 | 99.71 |
| ERR3148165 | 407_15S2 | 2124609 | 122.83 | 127 | 0.9631 | 99.43 |
| ERR3148212 | 408_15S3 | 856829 | 100.36 | 102 | 0.9785 | 99.59 |
| ERR3148166 | 421_14S4 | 525275 | 29.341 | 29 | 0.9395 | 99.43 |
| ERR3148168 | 456_14S5 | 650999 | 36.32 | 36 | 0.955 | 99.73 |
| ERR3148213 | 480_14S11 | 735083 | 80.226 | 82 | 0.9747 | 92.18 |
| ERR3148214 | 482_14S10 | 370909 | 19.952 | 20 | 0.8833 | 98.59 |
| ERR3148220 | 528_14S6 | 3051327 | 186.17 | 189 | 0.9713 | 99.48 |
| ERR3148169 | 570_15S11 | 792017 | 42.604 | 40 | 0.9532 | 99.74 |
| ERR3148184 | 580_14S23 | 3358677 | 208.17 | 172 | 0.9664 | 99.85 |
| ERR3148185 | 588_14S24 | 3719569 | 218.66 | 222 | 0.9765 | 99.66 |
| ERR3148218 | 661_14S15 | 3264939 | 199.99 | 202 | 0.9785 | 99.76 |
| ERR3148217 | 743_14S13 | 4391976 | 269.01 | 277 | 0.9795 | 99.77 |
| ERR3148186 | 768_16S7 | 2639963 | 166.2 | 173 | 0.9725 | 99.92 |
| ERR3148187 | 774_16S16 | 3135515 | 197.07 | 205 | 0.9771 | 99.91 |
| ERR3148188 | 781_16S19 | 3236782 | 203.28 | 210 | 0.976 | 99.9 |
| ERR3148189 | 801_16S13 | 3519134 | 220.25 | 229 | 0.977 | 99.92 |
| ERR3148190 | 815_14S20 | 3440178 | 215.55 | 221 | 0.9778 | 99.91 |
| ERR3148191 | 832_14S25 | 3287567 | 197 | 196 | 0.9796 | 99.83 |
| ERR3148192 | 882_16S17 | 2659929 | 167.71 | 175 | 0.9711 | 99.91 |
| ERR3148193 | 906_16S5 | 1750261 | 110.42 | 113 | 0.9734 | 99.9 |
| ERR3148216 | 916_14S10 | 3296541 | 206.33 | 205 | 0.9707 | 99.58 |
| ERR3148194 | 971_16S14 | 3939369 | 243.85 | 251 | 0.9772 | 99.88 |
| ERR3148154 | MSM_S9 | 334031 | 32.549 | 33 | 0.9516 | 92.64 |
